# Vascular Complication After Revision of Total Knee Arthroplasty (R-TKA): A Case of Popliteal Branch Pseudoaneurysm Successfully Treated with Embolization—A Case Report and Mini Review of the Literature

**DOI:** 10.3390/jcm15052079

**Published:** 2026-03-09

**Authors:** Karolina Zalewa, Piotr Piech, Karolina Nieoczym, Maciej Kozioł, Agnieszka Tomczyk-Warunek, Michał Sojka, Jacek Gągała, Maciej Szmygin, Ewa Tomaszewska, Jaromir Jarecki

**Affiliations:** 1 Department of Interventional Radiology and Neuroradiology, Medical University of Lublin, Jaczewskiego 8, 20-954 Lublin, Poland; zalewa.karolina@gmail.com (K.Z.); karolinanieoczym1@gmail.com (K.N.); maciej.szmygin@umlub.edu.pl (M.S.); 2 Department of Correct, Clinical and Imaging Anatomy, Medical University of Lublin, 20-954 Lublin, Poland; 3 Department of Traumatology, Orthopedics and Rehabilitation, Medical University of Lublin, Jaczewskiego 8, 20-954 Lublin, Poland; maciej.koziol@umlub.edu.pl (M.K.); jacek.gagala@umlub.edu.pl (J.G.); jaromir.jarecki@umlub.edu.pl (J.J.); 4 Department of Vascular Surgery, Medical University of Lublin, Jaczewskiego 8, 20-954 Lublin, Poland; michal.sojka@umlub.edu.pl; 5 Department of Animal Physiology, Faculty of Veterinary Medicine, University of Life Sciences in Lublin, Akademicka 13, 20-912 Lublin, Poland; ewa.tomaszewska@up.edu.pl

**Keywords:** total knee arthroplasty (TKA), revision knee arthroplasty (R-TKA), popliteal artery branch pseudoaneurysm, duplex ultrasound, computed tomography angiography (CTA), embolization

## Abstract

**Background:** Vascular injury after total knee arthroplasty (TKA) is rare but may be limb-threatening. Popliteal artery branch pseudoaneurysm is an uncommon complication that can present with nonspecific symptoms, potentially mimicking postoperative hematoma, infection, or deep venous thrombosis (DVT). **Case presentation:** A 79-year-old man underwent primary left TKA for advanced osteoarthritis (OA). Seven months later, he sustained a low-energy fall closed reduction and bracing were implemented. Due to recurrent episodes of instability with spontaneous self-reduction, a constrained revision TKA (R-TKA) was performed. Eighteen days after revision, the patient was readmitted because of persistent pain-related functional impairment. Duplex Doppler ultrasonography revealed a partially thrombosed pseudoaneurysm measuring 33 × 37 mm arising from a popliteal/genicular branch. Computed tomography angiography (CTA) confirmed a partially thrombosed pseudoaneurysm with a contrast-filled component within a larger periarticular fluid collection. This suggested a second, smaller pseudoaneurysm along the feeding vessel; hemarthrosis and soft-tissue edema were also present. After multidisciplinary evaluation, selective catheter angiography via left common femoral access was performed, and the injured branch was occluded using coil embolization combined with n-butyl cyanoacrylate tissue adhesive. Completion angiography demonstrated successful exclusion of the pseudoaneurysm without complications. **Conclusions:** Delayed pseudoaneurysm of a popliteal artery branch should be considered after revision TKA in patients with atypical swelling, hemarthrosis, or disproportionate pain. Duplex ultrasound and CTA are complementary diagnostic tools, and endovascular embolization provides a minimally invasive, effective, and low-morbidity treatment option when the lesion involves a branch vessel.

## 1. Introduction

### 1.1. Total Knee Arthroplasty (TKA) and Revision Knee Arthroplasty (R-TKA)

Total knee arthroplasty (TKA) is an effective treatment option for patients with advanced knee osteoarthritis (OA) [1]. Epidemiological studies predict that by 2040, approximately 78.4 million adults will be affected by OA, thereby increasing the number of primary TKAs performed. It is estimated that by 2050, the number of TKAs will rise by approximately 143% [2]. Although studies have demonstrated the high effectiveness of TKA in OA management, implant survivorship exceeds 90%. However, despite this, a substantial proportion of patients still require repeat procedures—referred to as revision TKA (R-TKA) [3,4]. Epidemiological data published by Laver et al. (2025) [5] estimated that 1–20% of patients undergo R-TKA, indicating considerable variability across patient cohorts. The most common indications for R-TKA include infection, mechanical complications, and dislocation [5]. The increasing volume of primary TKA will therefore be accompanied by a rise in the number of R-TKAs. Epidemiological data indicate that the number of revision procedures (R-TKA) will increase by 600% between 2005 and 2030 [2]. Importantly, R-TKA is associated with a higher risk of complications, longer hospital stays, and increased mortality compared with primary TKA. In primary TKA, overall patient survival at 15-year follow-up is reported to be 90–95%, whereas after R-TKA, patient survival drops to 71–80% at a shorter 10-year follow-up, indicating increased mortality among patients undergoing R-TKA [4,5].

### 1.2. Vascular Complications—Popliteal Artery Pseudoaneurysm (PPA)

Vascular complications (e.g., thrombosis, arteriovenous fistula formation, aneurysm, or pseudoaneurysm) are rare but carry a serious risk of limb ischemia, hemorrhage, and nerve compression [6]. A pseudoaneurysm, which involves disruption of the arterial wall with subsequent formation of an aneurysmal sac, may result from direct vessel injury, mechanical stretching, or thermal injury caused by bone cement [7]. It may present acutely or with delayed symptoms, including swelling, pain, an abnormally pulsatile mass, and hemarthrosis [8].

PPA is rare after TKA. Epidemiological data indicate an incidence of 0.0095% to 0.088% among patients [6,7]. However, PPA is the most common vascular injury associated with TKA [9]. Sundaram et al. (2020) reported that PPA accounted for as many as 45% of vascular injuries resulting from TKA [6]. In patients after R-TKA, as well as in the setting of infection and fractures, the risk of vascular complications, including arterial injury, is higher [10].

The most commonly used imaging modalities for diagnosing PPA include Doppler ultrasonography and computed tomography angiography (CTA). Magnetic resonance angiography (MRA) is also used occasionally. However, Doppler ultrasonography is the most frequently employed method. It provides excellent visualization of the disrupted arterial wall and enables detection of blood flow outside the vessel lumen [11]. Early diagnosis of PPA is crucial because failure to intervene promptly in the setting of reduced or obstructed blood flow may lead to nerve injury, limb loss, and even patient death [12,13,14].

### 1.3. Treatment of PPA

Based on the current literature, all pseudoaneurysms should be treated [15]. At present, conservative, surgical, or radiological management may be used (ultrasound-guided approaches, endovascular treatment, thrombin injection, or ultrasound-guided compression of the pseudoaneurysm). Conservative management (monitoring) is used only in cases of small, asymptomatic iatrogenic pseudoaneurysms, which may undergo spontaneous thrombosis [16,17].

Traditionally, pseudoaneurysms were treated surgically. However, this treatment has drawbacks, such as the need for general anesthesia and prolonged hospitalization, and in patients with comorbidities, it may be associated with impaired wound healing [15]. In the surgical treatment of PPA, repeat surgery in patients after TKA or R-TKA may entail a risk of infection of the recently implanted prosthesis. Moreover, surgical management of these pseudoaneurysms often requires harvesting a venous graft from the contralateral limb, which increases the risk of postoperative complications [18].

Therefore, over recent years, radiological methods have gained popularity and are currently the most commonly used approaches for treating pseudoaneurysms. Radiological treatment modalities include ultrasound-guided compression repair (UGCR), thrombin injection, and endovascular treatment [15]. UGCR is applied in smaller pseudoaneurysms associated with limited disruption of the vessel wall. This technique is most often used for pseudoaneurysms of superficial vessels, such as the brachial, axillary, and femoral arteries [18,19].

In the treatment of PPA, endovascular therapy is increasingly used and primarily based on two techniques: stent placement and embolization [15,20]. When selecting an endovascular strategy, the pseudoaneurysm size and the presence of collateral circulation should be assessed. If collateral supply is absent, embolization can be performed with coils, N-butyl-2-cyanoacrylate (glue), or detachable balloons [21]. In contrast, when collateral circulation is present, stent placement is used. In PPA management, stent-based treatment is more commonly employed and involves deploying a stent at the site of the arterial defect [7,9,11,14,18,22,23,24].

The aim of this study was to present a case of a patient diagnosed with a PPAafter R-TKA, successfully treated with endovascular embolization. A second aim was to emphasize the importance of interdisciplinary collaboration among specialists and departments to enable rapid, effective management of complex clinical conditions.

## 2. Case Description

### 2.1. Primary Arthroplasty of the Left Knee

#### 2.1.1. Medical History and Clinical Examination

A 79-year-old male patient (weight: 90 kg, height: 180 cm, body mass index (BMI): 27.78 kg/m^2^) was admitted due to pain-related functional impairment of the left knee. The symptoms were a consequence of advanced osteoarthritic changes that had been progressing for more than a decade. Over the preceding 5 years, symptoms had intensified despite regular use of non-steroidal anti-inflammatory drugs (NSAIDs). To alleviate symptoms, the patient had received multiple intra-articular steroid injections into the left knee at another center (at least three injections). The patient also underwent physiotherapy. However, despite treatment, his physical function deteriorated. Pain severity increased and was rated by the patient as 9/10 on the visual analog scale (VAS).

Physical examination of the left knee revealed enlarged joint contours without evidence of increased intra-articular effusion, with pain-related dysfunction involving all three compartments. Active and passive range of motion was 5–5–95°. Sagittal-plane stability was preserved, whereas coronal-plane instability was present. Valgus deformity of the knee measured 15°. The patella was immobile in the mediolateral direction. Peripheral sensation, perfusion, and limb function were preserved. The patient ambulated with the assistance of two forearm crutches.

The patient also had comorbidities, including arterial hypertension, nephrolithiasis, diabetes mellitus, and cardiac arrhythmia.

#### 2.1.2. Imaging Studies

**Figure 1:**
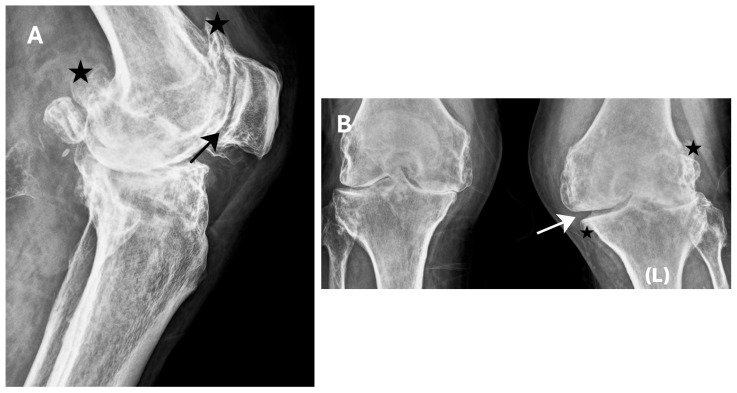
X-rays of the knee joints. (**A**) Lateral X-ray of the left knee joint. (**B**) Standing anteroposterior X-ray of both knee joints. Advanced degenerative changes. Significant valgus deformity. The joint space (white arrow) is significantly narrowed and asymmetric between the medial and lateral sides. Numerous osteophytes (black asterisks). The patellofemoral joint (black arrow) is significantly narrowed.

Anteroposterior (standing) and lateral radiographs (X-rays) of the knee were obtained (Figure 1). The X-rays showed severe OA in both knee joints. Massive osteophytes at the edge of the joint space (Figure 1A). Left knee joint valgus deformed. Osteophytes at the edge of the joint space. Closure of the joint space on the lateral side (Figure 1B).

**Figure 2:**
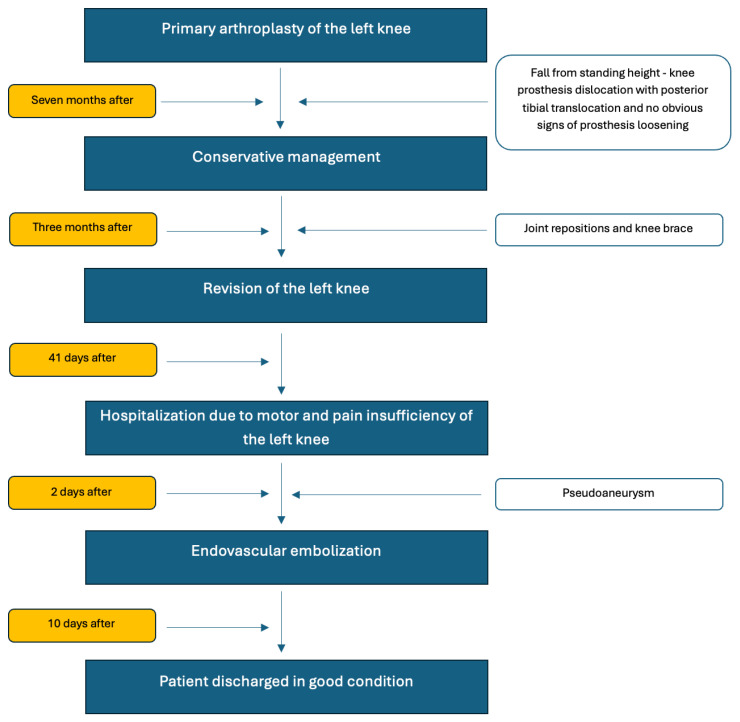
Treatment flow chart. The patient underwent primary total knee replacement of the left knee. Seven months later, the patient fell from a standing height. This resulted in a dislocation of the knee prosthesis with posterior tibial translocation, without any clear signs of prosthesis loosening. The patient received conservative treatment—repositioning and a knee brace. During follow-up visits, the patient reported additional falls from standing height (at least three episodes), after which symptoms of dislocation occurred with spontaneous self-reduction. The patient underwent revision surgery of the left knee. 41 days after discharge, the patient presented due to impaired mobility and pain in the left knee. The patient was diagnosed with a popliteal branch pseudoaneurysm. Embolization was performed.

Based on the clinical and imaging assessment, the patient was qualified for primary TKAof the left knee. However, the patient was disqualified at the initial scheduled date. During preoperative admission to the orthopedic ward, bradycardia and a rise in blood pressure to >200 mmHg were observed. A cardiology work-up (echocardiography, Holter monitoring, and ECG) was ordered, based on which a Biotronik DDD pacemaker was implanted at the Department of Cardiology. Anticoagulant therapy was also initiated (rivaroxaban 20 mg). Three months later, primary TKA of the left knee was performed (Figure 2).

#### 2.1.3. Surgical Procedure

The patient was positioned supine. A pneumatic tourniquet was applied to the left thigh at the level of the proximal one-third of the femur and inflated to 320 mmHg. Standard skin preparation and sterile draping were performed. A straight midline skin incision approximately 20 cm in length was made. TKA was performed through a standard medial parapatellar approach (medial arthrotomy). After exposure of the joint, extensive osteophytes were removed, particularly in the medial femoral condyle (MFC) region. Hypertrophic synovium was also excised, and a large loose body was removed from the posterior recess of the joint. Following femoral and tibial preparation according to the recommended technique, a Triathlon Stryker PS prosthesis was implanted (femur size 7, tibia size 7, 9-mm insert). The tourniquet was released after 1 h and 50 min. After its release, 1 g of tranexamic acid (Exacyl) was administered intravenously, according to the department protocol. The wound was closed in layers and covered with a soft dressing.

**Figure 3:**
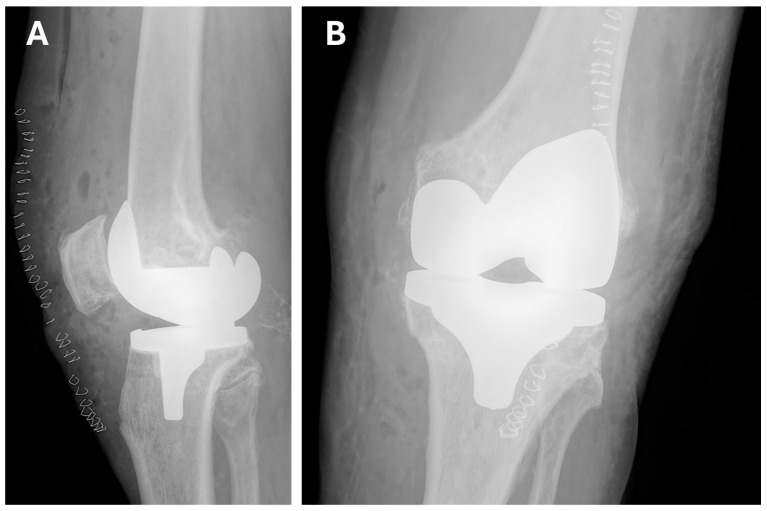
Postoperative X-ray of the left knee joint—day 1 after TKA. (**A**) Lateral plane. (**B**) Anteroposterior (AP) plane. The correct positioning of the femoral and tibial components is visible. The mechanical axis of the joint has been restored.

Twelve hours postoperatively, thromboprophylaxis was initiated with enoxaparin (Neoparin) at a therapeutic dose of 80 mg twice daily. Pain was controlled with NSAIDs. On postoperative day 1, follow-up X-rays were obtained (Figure 3). The patient was then mobilized on postoperative day 1 using a high walker. Early postoperative rehabilitation was implemented during hospitalization. Passive exercises of the left lower limb were performed using a continuous passive motion device (CPM) (DJO Global, Lewisville, TX, USA). On postoperative day 3 after TKA, the patient began ambulating with forearm crutches. The patient was discharged home on postoperative day 8 in good condition, with a recommendation to continue anticoagulant therapy (Clexane 840 mg, 1 prefilled syringe twice daily subcutaneously) for 30 days. After that period, anticoagulant therapy with rivaroxaban was restarted. Continuation of the exercises learned during hospitalization was also advised, along with further follow-up at the outpatient clinic.

### 2.2. Conservative Management

#### 2.2.1. Medical History and Clinical Examination

Seven months after primary TKA of the left knee, the patient presented to the emergency department. The day before, he had fallen in the bathroom from standing height, sustaining forced knee flexion with valgus stress. Clinical examination revealed marked enlargement of the joint contour with signs of increased joint effusion, deformity, and tenderness. Range of motion was limited by pain.

#### 2.2.2. Imaging Studies

**Figure 4:**
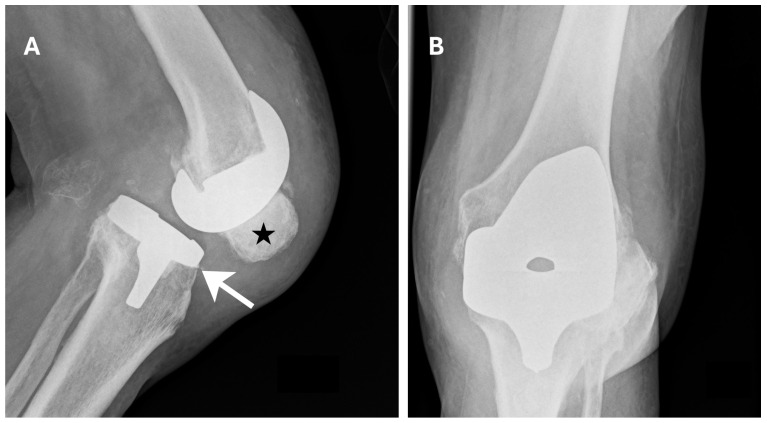
Postoperative X-ray seven months after TKA—left knee dislocation. (**A**) Lateral plane. (**B**) Anteroposterior (AP) plane. The lateral view shows joint instability in the form of posterior tibial dislocation (white arrow). Note the low position of the patella (black asterisk). The prosthetic components show no obvious signs of loosening.

Plain x-ray demonstrated dislocation of the left knee endoprosthesis with posterior translation of the tibia, without clear evidence of component loosening (Figure 4). Imaging studies confirmed dislocation of the left knee, a substantial amount of intra-articular fluid, lateralization of the patella, and an irregular calcification measuring approximately 30 mm in the popliteal fossa. No evidence of prosthetic loosening was identified.

**Figure 5:**
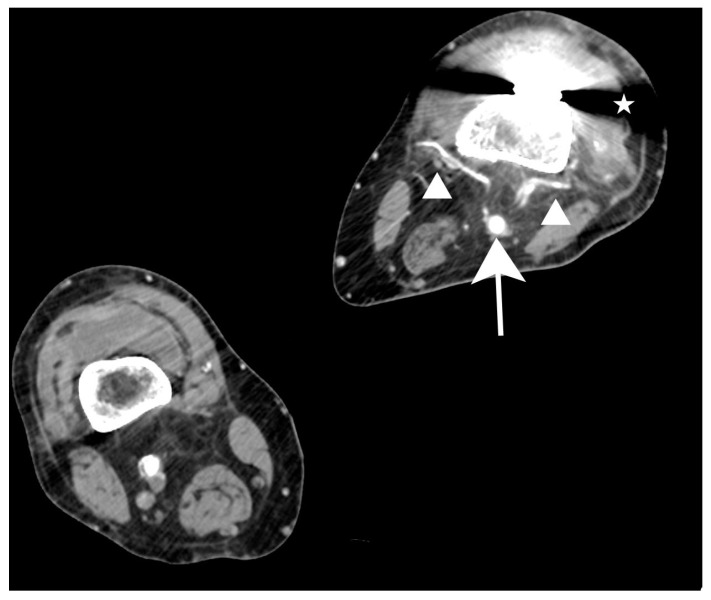
Representative CTA image of the knee joints after dislocation of the left joint—performed on the day of readmission to the hospital. Adequate contrast opacification of the popliteal artery (white arrow) and the arterial branches forming the genicular anastomoses (white arrowheads) is visible. Artifacts from the knee prosthesis (white asterisk) limit the assessment, but no contrast leakage outside the vessels was observed.

On CTA, lack of contrast opacification was noted in the distal segment of the left anterior tibial artery (at the level of the ankle joint) and the left dorsalis pedis artery, as well as in the distal segment of the medial branch of the left plantar artery. In the visible range, the main arteries of both lower limbs were otherwise appropriately opacified. The lack of contrast opacification in the aforementioned arteries was consulted with a vascular surgeon and considered a symptom of chronic lower limb ischemia, unrelated to the dislocation of the knee prosthesis (Figure 5).

**Figure 6:**
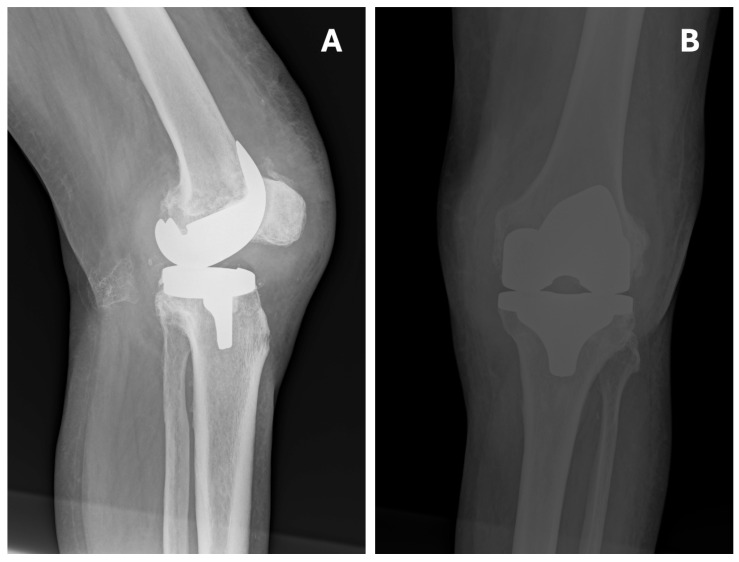
Postoperative X-ray after repositioning. (**A**) Lateral plane. (**B**) Anteroposterior (AP) plane. The correct axis is presented in both the AP and lateral views.

Based on the clinical examination and imaging findings, the patient was diagnosed with a traumatic injury to the medial collateral ligament (MCL). Conservative management was implemented, consisting of reduction of the knee joint and application of a knee brace (Figure 6). Outpatient follow-up at the trauma and orthopedic surgery clinic was recommended. The patient was discharged from the hospital in good condition after 4 days, with recommendations to use anticoagulant therapy and NSAIDs as needed for pain.

### 2.3. Revision of the Left Knee

During follow-up visits, the patient reported additional falls from standing height (at least three episodes), after which symptoms of dislocation occurred with spontaneous self-reduction. The patient did not use the knee brace at home. He was therefore qualified for revision surgery of the left knee.

Three months after the fall, the patient was admitted to the hospital for R-TKA of the left knee. However, the procedure was postponed because the required rivaroxaban washout period had not been observed. Three months later, revision surgery was performed.

#### Surgical Procedure

The patient was positioned supine. A pneumatic tourniquet was applied to the left thigh at the level of the proximal one-third of the femur and inflated to 320 mmHg. Standard skin preparation and sterile draping were performed. A straight skin incision approximately 20 cm in length was made along the previous scar. Synovial fluid was aspirated for culture. The procedure was performed through a standard midline approach (medial parapatellar arthrotomy). Intraoperatively, extensive damage to the MCL and the soft tissues of the medial compartment was identified. After exposure, the Triathlon Stryker PS prosthesis was removed. Subsequently, a constrained revision prosthesis was implanted: Triathlon MRH (femoral component: Large; 4 mm offset adapter; stem 80 mm/19 mm; tibial component: plateau Large; stem 15/80 mm; constrained MRH insert 13 mm). During prosthesis implantation, there was no need for extensive posterior capsular release. Tibial preparation and stem reaming were uneventful. The tourniquet was released after 1 h 35 min. After its release, no excessive bleeding was observed. 1 g of tranexamic acid (Exacyl) was administered intravenously, according to the department’s protocol. The wound was covered with a soft dressing.

**Figure 7:**
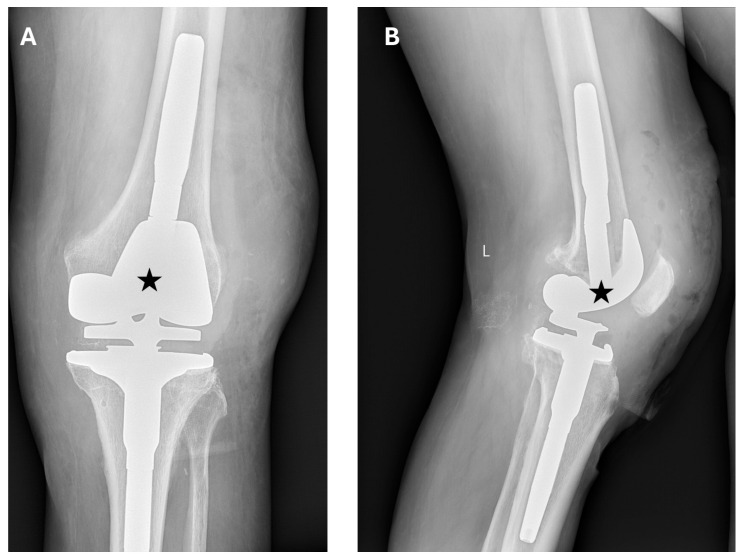
Postoperative X-ray—1 day after R-TKA. (**A**) Anteroposterior (AP) plane. (**B**) Lateral plane. The correct positioning of the prosthesis components (black asterisk) is visible.

Twelve hours after surgery, thromboprophylaxis was initiated with enoxaparin (Neparin). Pain was controlled with NSAIDs. On postoperative day 1, follow-up X-rays were obtained (Figure 7). The patient was then mobilized on postoperative day 1 using a high walker. Early postoperative rehabilitation was implemented during hospitalization. Passive exercises of the left lower limb were performed using a CPM (DJO Global, Lewisville, TX, USA). On postoperative day 3 after TKA, the patient began ambulating with forearm crutches. During postoperative care and rehabilitation, no signs of hematoma formation were observed at the surgical site. The patient was discharged from the hospital after 15 days in good condition, with recommendations similar to those after primary TKA.

### 2.4. Pseudoaneurysm

#### 2.4.1. Medical History and Clinical Examination

After discharge, in the late postoperative period (41 days after the revision surgery), the patient was readmitted due to persistent pain-related functional impairment of the knee.

On admission, the left lower limb was warm with preserved distal perfusion. Capillary refill was <2 s. Peripheral pulses were assessed and were palpable at the femoral level and distally at the posterior tibial and dorsalis pedis arteries. No clinically relevant pulse deficit or side-to-side asymmetry was identified, and there were no features of acute limb ischemia (no pallor, cyanosis, coldness, or rest pain).

Local inspection of the knee revealed periarticular swelling, more pronounced laterally and toward the popliteal region, consistent with the subsequently confirmed pseudoaneurysm and associated hemarthrosis. The overlying skin was intact, without erythema, increased local temperature, or signs of wound complication. On palpation, the mass was tender and deep-seated. A subtle pulsatile component could be appreciated without active bleeding. No audible bruit was detected over the popliteal fossa.

Neurological status was intact: light-touch sensation in the foot was preserved, and active ankle/toe motion was maintained. There were no symptoms suggestive of tibial or peroneal nerve compromise. Overall, the findings were consistent with a hemodynamically stable branch-vessel lesion without compromise of the main arterial axis, supporting further vascular imaging and endovascular management.

#### 2.4.2. Imaging Studies

**Figure 8:**
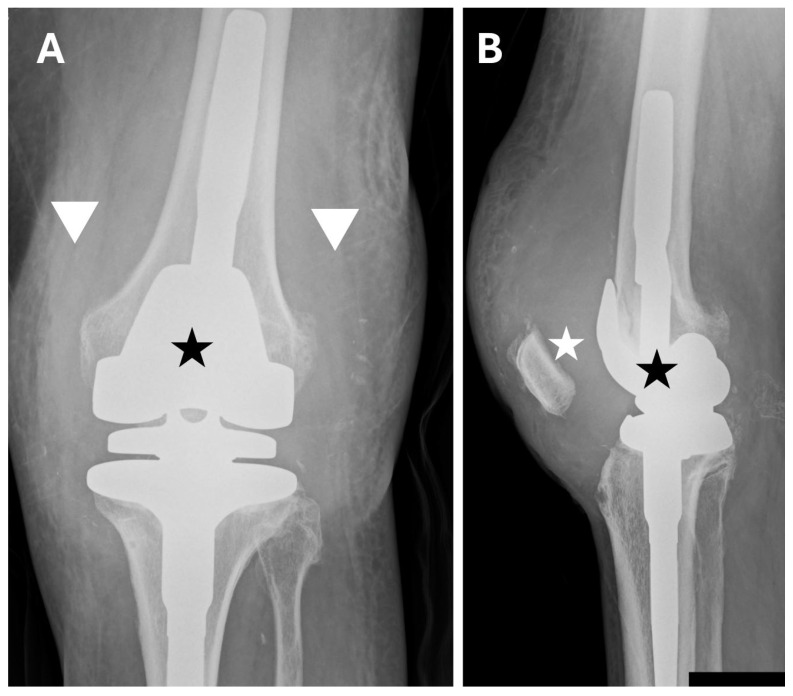
X-ray taken upon readmission after R-TKA. (**A**) Anteroposterior (AP) plane. (**B**) Lateral plane. AP and lateral views show correct alignment of the prosthetic components (black asterisks). The AP view shows significant widening of the joint outline (white arrowheads). Significant joint effusion causing an increase in the distance between the patella and the femoral component of the prosthesis (white asterisk).

Initially, an X-ray of the operated knee was performed, which revealed significant expansion of the soft-tissue outline, a large joint effusion, and no obvious signs of loosening of the prosthetic components (Figure 8).

**Figure 9:**
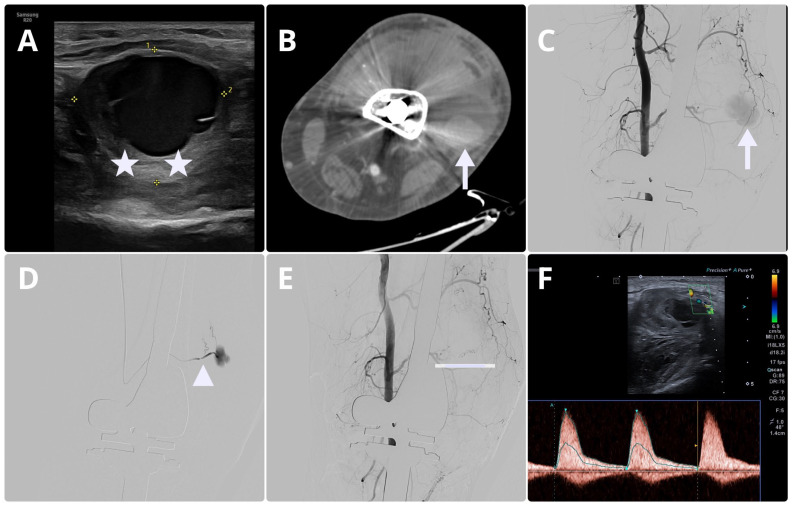
Baseline B-mode ultrasound demonstrated a partially thrombosed pseudoaneurysm (thrombosis visible in the lower part of the lesion—white asterisks) originating from the lateral branch of the popliteal artery (**A**). Doppler modality showed slow filling of the lesion. The diagnosis was further confirmed by computed tomography angiography (CTA), which disclosed the presence of a partially filled lesion at the distal part of the branch of the genicular artery (white arrow) (**B**). Initial angiography via a catheter placed in the popliteal artery revealed a pseudoaneurysm arising from the superior genicular artery with delayed filling of contrast (**C**) (white arrow). A microcatheter was then positioned, and embolization was performed (**D**) (white triangle—tip of the microcatheter). Control Digital subtraction angiography (DSA) confirmed complete occlusion of the lesion with no residual filling (white line—coils) (**E**). Follow-up ultrasound showed further thrombosis and reduction in size, with preserved flow in the proximal segment of the artery (**F**).

Doppler ultrasonography was performed and revealed a partially thrombosed pseudoaneurysm (33 × 37 mm) arising from a lateral branch of the popliteal artery. The examination assessed the arteries of the left lower limb, including the common, superficial, and deep femoral arteries, the popliteal artery, and the anterior and posterior tibial arteries. On the lateral aspect of the left knee, a partially thrombosed pseudoaneurysm of one of the popliteal artery branches measuring approximately 33 × 37 mm was visualized. No other significant morphological abnormalities were identified in the evaluated arteries. Blood flow parameters were normal, with a high-resistance, triphasic waveform. Probable explanation for the preservation of normal blood flow in the genicular artery is the fact that the pseudoaneurysm was located distally from the genicular artery. Soft-tissue edema was noted in the region of the left knee joint (Figure 9A).

Based on these findings, CTA of the left knee was ordered. The examination demonstrated a fluid-density area measuring approximately 55 × 48 mm along the lateral contour of the distal metaphysis of the left femur, with a contrast-filled focus within it measuring approximately 31 × 24 mm. One of the branches of the left popliteal artery was observed to course toward the described lesion. The CTA appearance suggested a partially thrombosed pseudoaneurysm along its course. In addition, in the proximal segment of the small feeding artery, a focal enlargement measuring approximately 6 × 6.5 mm was identified, consistent with a possible second, smaller pseudoaneurysm. A large amount of heterogeneous, dense content (most likely partially hemorrhagic) was also observed within the left knee joint, with small calcifications present. Edematous changes of the soft tissues of the left lower limb were noted, particularly at the level of the knee joint (Figure 9B).

The patient was qualified for endovascular embolization. The procedure was performed 2 days after readmission to the hospital.

#### 2.4.3. Procedure Description

The procedure was performed under local anesthesia with 10 mL of 2% lidocaine. Vascular access was obtained via percutaneous puncture of the left common femoral artery (L CFA), and a 5F introducer sheath was inserted. Digital subtraction angiography (DSA), performed using Visipaque 320 (radiation exposure: RP 158 mGy), total time of fluoroscopy 10.5 min, total dose of contrast media—50 mL) demonstrated opacification of the pseudoaneurysm via a feeding branch originating from the genicular artery (GA) (Figure 9C).

The feeding vessel was selectively catheterized, and distal occlusion was achieved using embolization coils (Nester—2 coils 2 × 5mm) followed by injection of tissue adhesive (Glubran2 mixed with Lipiodol, 30% of glue), resulting in exclusion of the pseudoaneurysm from the circulation (Figure 9D). Completion DSA confirmed effective pseudoaneurysm exclusion with preserved flow in the remaining evaluated vessels and no procedure-related complications (Figure 9E,F). Hemostasis at the access site was achieved with manual compression and a pressure dressing, which was recommended for removal after at least 4 h. The patient, in good general condition, was transferred back to the orthopedic department. There was no need to modify anticoagulation after embolization.

Following the procedure, the patient remained in the hospital for 10 days. During this time, the left knee joint was regularly monitored, and the patient received appropriate rehabilitation. A gradual reduction in knee circumference was observed. A bedside ultrasound performed in the department of orthopedics confirmed complete embolization of the pseudoaneurysm (5 days after procedure—Figure 9F). The patient was discharged home in good general condition. Continuation of the exercises learned during hospitalization was recommended, along with further follow-up at the outpatient clinic. Due to cardiological indications, anticoagulant therapy with rivaroxaban (20 mg daily) was continued.

## 3. Discussion

Vascular injuries associated with TKA are exceptionally uncommon, with an estimated incidence ranging from 0.03% to 0.2% of procedures [25]. As a result of these injuries, pseudoaneurysms may occur. Pseudoaneurysms develop when blood extravasates from an injured vessel and is contained within a sac composed primarily of fibrin and platelets [26]. In the setting of pseudoaneurysm formation after TKA, the presence of prosthetic components and the constrained periarticular anatomy increase the risk of inadvertent vascular injury [27]. Epidemiological data indicate that the incidence of PPA after TKA is 0.0095–0.088% of patients [6,7]. In our case, the history of multiple interventions (reduction and subsequent revision surgery) may have contributed to cumulative vessel wall stress and microtrauma. Additionally, one can speculate that medial compartment soft-tissue damage and constrained prosthetic geometry might have altered local biomechanics and stress distribution in the vicinity of the vascular structures. Clinically, reported presentations range from swelling and pain to hemarthrosis, and may even mimic deep venous thrombosis (DVT) [27]. Our case was consistent with these observations.

With regard to imaging, duplex Doppler ultrasonography is often the first-line modality because it is safe and noninvasive, and it enables detection of flow within the pseudoaneurysm cavity [28]. CTA provides detailed anatomic information, allows precise localization, and offers a broader overview of surrounding structures, although metallic artifacts may reduce image quality.

Traditionally, open surgical repair (ligation, resection, grafting) was the mainstay of treatment for popliteal pseudoaneurysms [14,29,30,31,32]. However, endovascular and percutaneous techniques have become preferred in many cases due to lower morbidity, faster recovery, and the ability to combine diagnosis and treatment within a single session [33]. Available options include coil embolization, tissue adhesive, thrombin injection, covered stent placement, or combined approaches. In the post-TKA setting, coil-based strategies can be effective while sparing the main vessel when the lesion arises from a branch. Our patient was discussed at a multidisciplinary team meeting involving an orthopedic surgeon, a vascular surgeon, and an interventional radiologist. The least invasive options initially considered were ultrasound-guided interventions, including thrombin injection and ultrasound-guided compression. Both methods are recognized therapeutic options for selected peripheral pseudoaneurysms; however, their effectiveness in the knee region remains controversial [9]. In our case, both methods were also ruled out due to the lesion’s wide neck and lateral location, which might preclude effective compression. Open surgical repair was rejected because of the patient’s history of multiple prior procedures in the affected area, likely associated with significant fibrosis and altered local anatomy. Endovascular stent placement, although highly effective for PPA [9,22,24], was also excluded due to the small diameter of the parent artery [24].

In our patient, the pseudoaneurysm involved the superior genicular artery, a vessel commonly occluded during genicular artery embolization procedures without increased risk of clinically significant ischemia due to the presence of an anastomotic network between genicular arteries [34]. The importance of these collaterals and their impact on embolization procedures in the knee region was presented by Amin et al. [35]. Also, based on the studies included in Table 1, it can be concluded that pseudoaneurysm embolization is most commonly used to treat branches of the genicular artery [28,36,37]. However, based on this table, it can also be concluded that the most frequently reported treatment was in patients after TKA, not R-TKA [28,36,37]. In our case, the procedure was performed on a patient after R-TKA. Consequently, coil and glue embolization was performed successfully, resulting in effective exclusion of the pseudoaneurysm without complications.

## 4. Conclusions

This case underscores that a pseudoaneurysm of a popliteal branch may complicate revision TKA even many weeks after implantation. Recognition requires a high index of suspicion, especially in patients with atypical joint swelling or pain. Duplex ultrasound and angiographic imaging are complementary tools for diagnosis. Endovascular embolization offers a minimally invasive, effective, and low-morbidity option for excluding the lesion. In our opinion, the novelty of the present case arises from a combination of several distinctive features. First, while the majority of published reports describe pseudoaneurysms involving the main trunk of the popliteal artery, our patient developed a lesion originating from a distal segment of a small branch arising from the popliteal artery. This unusual branch-vessel location significantly increased the technical complexity of the endovascular procedure, particularly with respect to selective catheterization and embolization. Second, most available reports describe early symptom onset, typically occurring within days following the inciting surgical procedure. In contrast, our patient presented late in the postoperative period, which may have delayed clinical suspicion and diagnosis. Finally, although the diagnostic pathway itself was standard, the management strategy contributed additional novelty. The use of combined embolic materials (coils with tissue adhesive) appears to be less commonly reported in similar cases and allows effective exclusion of the pseudoaneurysm while preserving the parent vessel. Taken together, the uncommon distal branch origin, delayed clinical presentation, and combined embolization technique distinguish this case from previously published reports and underscore its clinical relevance.

## Figures and Tables

**Table 1:** Summary of previously reported cases of pseudoaneurysms after TKA or R-TKA.

References	TKA Type	Involved Vessel	Time to Presentation	Treatment
[9]	R-TKA	Popliteal artery	Several days	Covered stent
[36]	Primary TKA	Inferior lateral genicular artery	10–14 days	Coil embolization
[30]	Primary TKA	Inferior medial genicular artery	~4 weeks	Surgical treatment
[31]	Primary TKA	Superior medial genicular artery	Early postoperative	Surgical treatment
[25]	Primary TKA	Superior lateral genicular artery	Early postoperative	Coil embolization
[37]	TKA	Superior medial and lateral genicular arteries	Several days	Compression/embolization
[32]	1. Primary TKA (2 cases), 2. R-TKA (1 patient)	Popliteal artery/branches	Variable	1. Percutaneous embolization (2 patients), 2. Surgical treatment (1 patient).
[14]	Primary TKA	Popliteal artery	Several days	Surgical treatment
[28]	TKA	Popliteal + genicular arteries	Variable	1. Mainly embolization (5 cases), 2. Thrombosed without intervention (1 participant), 3. Surgical treatment (1 patient).
[24]	TKA	Various vascular complications	—	1. Stents (9 cases), 2. Endarterectomies (2 patients), 3. Thrombectomy (1 case), 4. Bypass (1 participant).
[22]	TKA	Popliteal artery injury	Early postoperative	Endovascular repair—Stent

Legend: TKA—total knee arthroplasty; R-TKA—revision total knee arthroplasty.

## Data Availability

All results are presented in this paper.

## Abbreviations

The following abbreviations are used in this manuscript:

TKA	Total knee arthroplasty
R-TKA	Revision knee arthroplasty
CT	Computer tomography
OA	Osteoarthritis
PPA	Popliteal artery pseudoaneurysm
UGCR	Ultrasound-guided compression repair
BMI	Body mass index
CTA	Computed tomography angiography
MRA	Magnetic resonance angiography
NSAIDs	Non-steroidal anti-inflammatory drugs
VAS	Visual analog scale
X-rays	Radiographs
MFC	Medial femoral condyle
CPM	Continuous passive motion device
MCL	Medial collateral ligament
L CFA	Left common femoral artery
DSA	Digital subtraction angiography
SFA	Superficial femoral artery
DVT	Deep venous thrombosis
